# Efficacy of elobixibat as bowel preparation agent for colonoscopy: Prospective, randomized, multi‐center study

**DOI:** 10.1111/den.14010

**Published:** 2021-05-24

**Authors:** Daisuke Yamaguchi, Hidenori Hidaka, Takuya Matsunaga, Takashi Akutagawa, Yuichiro Tanaka, Amane Jubashi, Yuki Takeuchi, Nanae Tsuruoka, Yasuhisa Sakata, Koichi Miyahara, Naoyuki Tominaga, Hiroharu Kawakubo, Ayako Takamori, Ryo Shimoda, Takahiro Noda, Shinichi Ogata, Seiji Tsunada, Motohiro Esaki

**Affiliations:** ^1^ Department of Gastroenterology National Hospital Organization Ureshino Medical Center Saga Japan; ^2^ Division of Gastroenterology Department of Internal Medicine Faculty of Medicine Saga University Saga Japan; ^3^ Department of Internal Medicine Karatsu Red Cross Hospital Saga Japan; ^4^ Department of Gastroenterology Saga‐ken Medical Centre Koseikan Saga Japan; ^5^ Department of Internal Medicine Imari‐Arita Kyoritsu Hospital Saga Japan; ^6^ Clinical Research Center Saga University Hospital Saga Japan

**Keywords:** bowel cleansing, bowel preparation, colonoscopy, elobixibat, tolerability

## Abstract

**Background and Aim:**

Elobixibat is a novel ileal bile acid transporter inhibitor. This study aimed to compare the efficacy, tolerability, and safety of the combination of elobixibat and 1 L of polyethylene glycol formulation containing ascorbic acid (PEG‐Asc) solution versus the combination of sodium picosulfate and 1‐L PEG‐Asc solution as bowel preparation for colonoscopy.

**Methods:**

This multi‐center, randomized, observer‐blinded, non‐inferiority study recruited 210 outpatients who were assigned to either the elobixibat plus 1‐L PEG‐Asc group (group A) or the sodium picosulfate plus 1‐L PEG‐Asc group (group B). The quality of the bowel cleansing level was assessed by the Boston Bowel Preparation Scale (BBPS) and compared the bowel cleansing level between the groups. Data regarding bowel preparation time, patients’ tolerability, and adverse events were also analyzed.

**Results:**

Data for 196 patients (99 in group A and 97 in group B) were analyzed finally. BBPS was comparable between group A and B (8.3 ± 0.9 vs. 8.3 ± 0.7; *P *= 0.88). Consequently, the adequate bowel preparation rate in groups A and B was 95.0% and 99.0%, respectively (−4.0%, 95% CI −9.3 to 1.5). Bowel preparation time in group A was similar to that in group B (348.2 ± 79.8 min vs. 330.8 ± 82.5 min; *P* = 0.13), whereas, sleep disturbance was significantly less frequent in group A than in group B (10.2% vs. 22.7%; *P *= 0.02).

**Conclusions:**

The combination of elobixibat and 1‐L PEG‐Asc can be considered an alternative bowel preparation for colonoscopy considering the equivalent bowel cleansing effect and less frequent sleep disturbance. The Japan Registry of Clinical Trials (jRCTs41180026).

## Background

Colonoscopy is the first choice modality for diagnosing colorectal diseases and it is well known that colonoscopy contributes to decreasing mortality in colorectal cancer.^1^ However, appropriate bowel preparation is crucial to improve the diagnostic ability of the procedure because insufficient colonic cleansing may impair the detection of even large polyps or masses, or hamper endoscopic manipulation, causing longer procedure times or increasing the risk of procedure‐related complications.^2^, ^3^, ^4^, ^5^, ^6^, ^7^, ^8^

Polyethylene glycol (PEG) solution has been commonly used for bowel preparation for colonoscopy.^9^ However, large amounts of lavage solution and unfavorable palatability sometimes hinder patients’ acceptance.^3^ To address these disadvantages, modified methods of bowel preparation using smaller volumes of PEG solution combined with ascorbic acid (Asc) or bisacodyl,^4^, ^8^, ^9^, ^10^ or with Asc plus sodium picosulfate^11^, ^12^, ^13^, ^14^ have been developed, and their better acceptability and favorable cleansing ability have been reported. On the basis of these results, bowel preparation using 1‐L of PEG‐Asc combined with sodium picosulfate has been generally used for bowel preparation for colonoscopy in Japan. However, patients sometimes complain of abdominal pain or sleep disturbance after oral administration of sodium picosulfate because the laxative is taken the night before ileocolonoscopy.

Elobixibat is a novel medication for chronic constipation that acts as a bile acid transporter inhibitor.^15^ By inhibiting resorption of bile acids in the terminal ileum, elobixibat increases the amount of bile acid in the colon, subsequently enhancing colonic motility and secretion.^16^ Actually, Nakajima et al. reported that once daily administration of 10 mg elobixibat significantly shortened the time to the first spontaneous bowel movements compared to placebo (8.2 h vs. 36.2 h, *P* < 0.001).^17^ Based on the result, we hypothesized that switching from picosulfate at night to elobixibat in the morning could promote defecation during daytime and reduce sleep disturbance on the day before colonoscopy.

In this study, we compared the efficacy, tolerability and safety of the combination of elobixibat and 1‐L PEG‐Asc solution with the combination of sodium picosulfate and 1‐L PEG‐Asc solution as bowel preparation for colonoscopy.

## Methods

### Patients and study design

This study was a prospective, multi‐center, randomized, single‐blind non‐inferiority trial in adult patients who were scheduled for elective colonoscopy. The study was conducted at five centers, namely Ureshino Medical Center, Karatsu Red Cross Hospital, Saga Medical Centre Koseikan, Saga University Hospital and Imari‐Arita Kyoritsu Hospital. This clinical trial was conducted in accordance with the Declaration of Helsinki and the guidelines of the Consolidated Standards of Reporting Trials (CONSORT). The study protocol was approved by the Institutional Review Board of the Shizuoka Cancer Center Hospital and Research Institute, and written informed consent was obtained from all patients. The trial was registered with the Japan Registry of Clinical Trials (jRCTs41180026) on 5 December 2018.

The enrolled subjects were adults aged ≥20 years who were scheduled for an outpatient colonoscopy from January 2019 to February 2020. The primary purpose of the patient's colonoscopy was a positive secondary fecal occult blood test or follow‐up after a colonic polypectomy. Patients were excluded if they had any of the following conditions: ileus, suspected bowel obstruction or toxic megacolon; prior abdominal or pelvic surgery; inflammatory bowel disease; advanced malignancy; severe liver damage (Child–Pugh classification Grade C); dementia or other cognitive disorders or hypersensitivity to either elobixibat, sodium picosulfate, or PEG solution. Female patients were excluded from the study if they were pregnant or breastfeeding.

**Figure 1:**
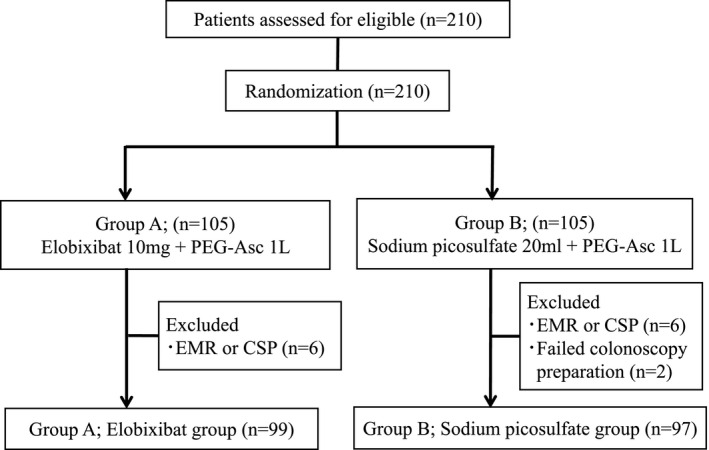
Study flow chart of the patient randomization and inclusion in the analyzed groups. CSP, cold snare polypectomy; EMR, endoscopic mucosal resection.

Patients who met the eligibility criteria were sequentially allocated into two groups using an internet‐based, random number generator (Fig. 1). Concealment of randomization was retained by personnel who were not involved in the colonoscopy procedure, the outpatient department or data collection and analysis.

### Bowel preparation for colonoscopy

**Figure 2:**
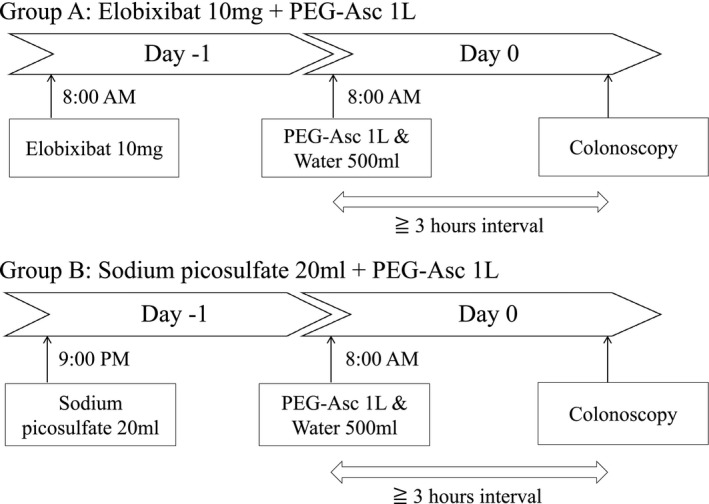
Bowel preparation protocols. Group A: elobixibat 10 mg + PEG‐Asc 1 L. Group B: sodium picosulfate 20 mL + PEG‐Asc 1 L. PEG‐Asc, polyethylene glycol formulation containing ascorbic acid.

Dietary restriction and avoidance of prokinetics and laxatives other than bowel preparation for 3 days prior to the colonoscopy were instructed to each patient. Patients were assigned to either elobixibat and PEG‐Asc group (group A) or sodium picosulfate and PEG‐Asc group (group B). On the day before colonoscopy, patients in group A took 10 mg of elobixibat (GOOFICE; Mochida Pharmaceutical Co., Ltd., Tokyo, Japan) at 8:00 AM, while those in group B consumed 20 mL of sodium picosulfate (Sodium Picosulfate Hydrate; Pfizer Japan Inc., Tokyo, Japan) with 100 mL of water at 9:00 PM. On the day of colonoscopy, patients in both groups started 1‐L PEG‐Asc solution (Moviprep; EA Pharma Co., Ltd., Tokyo, Japan) with 500 mL of water at 8:00 AM. Patients were instructed to take PEG‐Asc solution and water alternately at a rate of 500 mL every 30 min (Fig. 2).

The 500 mL of the PEG‐Asc solution with 250 mL of water was added if two nurses judged the stool was not clean. Patients were instructed to complete the administration of the lavage solution at least 3 h before colonoscopy.

### Assessment of colon cleansing

All endoscopists were instructed to take endoscopic images of each colonic segment that were representative of the colonic cleansing level when the scope was inserted to the caecum. The Boston Bowel Preparation Scale (BBPS) was used to assess the cleansing level of each colonic segment.^18^, ^19^ The detail of BBPS scoring was described in Appendix S1. More than poor (BBPS score 6 or higher) was considered adequate bowel preparation.

Endoscopic images were assessed by two endoscopists who were blinded to the type of bowel preparation, and the bowel cleansing grade was determined. Differences in the BBPS between the two endoscopists were resolved by consensus. Colonoscopy was performed by 16 endoscopists (11 specialists and 5 trainees) with a mean endoscopic experience of 12 (range 6–13) years. Specialists were defined as endoscopists who had Board Certified Fellow of the Japan Gastroenterological Endoscopy Society.

### Assessment of procedure‐related outcomes and tolerability

The investigator collected the data for the first defecation time and the number of stools after taking the medication on the day before colonoscopy. The first defecation time was defined as the time from taking the medication to the time of the first defecation. The investigator also collected data for the bowel preparation time on the day of colonoscopy, which was defined as the time from 8:00 AM until colonoscopy.

During the colonoscopy, cecal insertion time and withdrawal time, as well as colonoscopic findings, were recorded to assess the procedure outcome. When colonic lesions requiring endoscopic removal were found under colonoscopy, patients were generally recommended to undergo repeat colonoscopy at a later date to ensure fairness in testing times and adverse events.

The tolerability of bowel preparation was analyzed by assessing adverse events and palatability using a 5‐point scale questionnaire (Appendix S2).

### Study outcomes

The primary endpoint of the present study was to determine the difference in the colon cleansing efficacy between the two groups. The secondary endpoints were to assess the bowel preparation times and tolerability, procedure‐related outcomes, and adverse events of both groups.

### Statistical analysis

The sample size of this non‐inferiority study was based on previously published results and the preliminary results of adequate bowel cleansing level by sodium picosulfate and 1‐L PEG‐Asc solution at our hospital. We assumed that the overall rate of adequate bowel cleansing by sodium picosulfate and 1‐L PEG‐Asc solution would be approximately 85%.^12^, ^20^, ^21^ We hypothesized that a minimum 15% difference in the rates of adequate bowel cleansing between the two preparations would constitute a clinically meaningful difference. Since there was no pilot study on elobixibat, we set the same inferiority margin according to the previous inferiority study.^10^, ^22^ Considering a drop‐out rate of 18%, the total sample size required was calculated to be 210 (105 patients in each group), using the assumption of α = 0.025 and β = 0.20. The bowel cleansing level in group A would be considered non‐inferior to group B if the lower limit of 95% confidence interval (CI) for the treatment difference was greater than −15.0%. Treatment difference was calculated by subtracting the percentage of patients achieving adequate bowel preparation in group B from the value in group A, and the difference was described as the percentage with 95% CI.

We performed for per‐protocol (PP) and intention‐to‐treat (ITT) analyses sets. Categorical data were expressed as number (percentage), and the χ^2^ test was used to identify differences between the two groups. Numerical data were expressed as mean ± standard deviation, and Student’s *t*‐test was used to determine differences between the two groups. All statistical tests were two‐sided without adjustment for multiple comparisons. *P* values <0.05 were considered statistically significant for each test, and all statistical analyses were performed with JMP version 13.0.0 (SAS Institute Inc., Cary, NC, USA).

## Results

### Baseline characteristics

The flow chart of the study subjects is shown in Figure 1. Six patients in each group who underwent the procedure at the reference colonoscopy were excluded from the present study. Two patients in group B were excluded because of the failure of bowel preparation. A total of 196 subjects completed the study protocol and were included in the final PP analysis.

Table 1 compares the baseline characteristics between the two groups. None of the patient‐related factors differed between the two groups. In addition, no significant difference was found between the two groups regarding comorbidities as well as for the Charlson comorbidity score.

**Table 1:** Patients’ characteristics

	Group A	Group B	*P* value
Number of patients (*n*)	99	97	
Age (years)	64.8 ± 13.9	63.4 ± 13.3	0.45
Sex, male	48 (48.5%)	51 (52.6%)	0.57
BMI (kg/m^2^)	23.2 ± 4.0	22.5 ± 2.8	0.17
Alcohol drinking	48 (48.5%)	37 (38.1%)	0.15
Smoking	38 (38.4%)	34 (35.1%)	0.66
History of colonoscopy	46 (46.4%)	46 (47.4%)	1.00
Using laxative	5 (5.0%)	5 (5.2%)	1.00
Using sleeping pills	4 (4.0%)	4 (4.1%)	1.00
Using antithrombotic agents	15 (15.2%)	15 (15.5%)	1.00
Comorbidity			
Cardiovascular diseases	14 (14.1%)	17 (17.5%)	0.56
Cerebrovascular diseases	5 (5.0%)	9 (9.3%)	0.28
Chronic kidney diseases	3 (3.0%)	1 (1.0%)	0.62
Chronic liver damage	5 (5.0%)	5 (5.2%)	1.00
Diabetes mellitus	14 (14.1%)	7 (7.2%)	0.17
Hypertension	35 (35.4%)	40 (41.2%)	0.46
Malignant diseases	22 (22.2%)	15 (15.5%)	0.27
Charlson comorbidity score	1.4 ± 1.7	1.2 ± 1.2	0.38

BMI, body mass index. Results are presented as mean ± standard deviation or number of patients.

### Primary endpoint

The bowel cleansing grade between the two endoscopists was concordant in 190 out of 196 patients (96.9%). The grades were finally determined by consensus in the remaining six patients.

**Figure 3:**
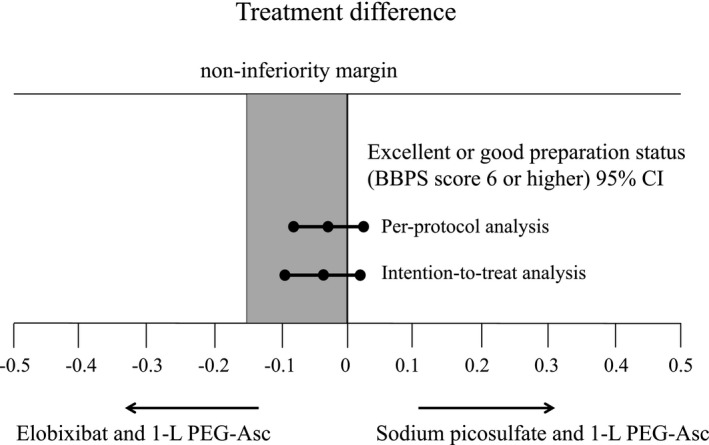
Treatment differences between bowel preparation with elobixibat and 1‐L PEG‐Asc versus with sodium picosulfate and 1‐L PEG‐Asc. The treatment difference in the excellent or good preparation status (BBPS score 6 or higher) was −4.0% (95% CI −9.3 to 1.5) in the per‐protocol analysis and −4.8% (95% CI −10.0 to 0.7) in the intention‐to‐treat analysis, falling within the 15% non‐inferiority margin. PEG‐Asc, polyethylene glycol formulation containing ascorbic acid.

Table 2 compares bowel cleansing level between the two groups based on PP and ITT analysis set. The number of patients achieving adequate bowel preparation was 94 (95.0%) patients in group A and 96 (99.0%) patients in group B. The difference was determined to be −4.0% (95% CI −9.3 to 1.5) in PP analysis set (Fig. 3), thus demonstrating non‐inferiority of bowel preparation of group A over group B.

**Table 2:** Colon cleansing level graded in accordance with the Boston Bowel Preparation Score and bowel preparation time

	Intention‐to‐treat analysis			Per‐protocol analysis		
	Group A	Group B	*P* value	Group A	Group B	*P* value
Number of patients (*n*)	105	105		99	97	
Colon cleansing						
BBPS, whole colon	8.3 ± 1.0	8.3 ± 0.7	0.66	8.3 ± 1.0	8.3 ± 0.7	0.66
BBPS, right colon	2.5 ± 0.5	2.5 ± 0.5	0.22	2.5 ± 0.5	2.5 ± 0.5	0.20
BBPS, transverse colon	2.9 ± 0.4	2.9 ± 0.3	0.16	2.9 ± 0.3	2.9 ± 0.3	0.20
BBPS, left colon	2.9 ± 0.5	2.9 ± 0.2	0.11	2.9 ± 0.5	2.9 ± 0.2	0.19
Preparation rating						
Excellent (BBPS 8–9)	87 (82.8%)	92 (87.6%)	0.12	82 (82.8%)	87 (89.7%)	0.21
Good (BBPS 6–7)	12 (11.4%)	10 (9.5%)		12 (12.1%)	9 (9.3%)	
Poor (BBPS 3–5)	6 (5.7%)	1 (1.0%)		5 (5.1%)	1 (1.0%)	
Inadequate (BBPS 0–2)	0 (0%)	0 (0%)		0 (0%)	0 (0%)	
First defecation time (h)	4.4 ± 5.5	6.7 ± 3.8	<0.001	4.5 ± 5.6	6.7 ± 3.2	<0.001
Number of defecation of the day before	3.2 ± 2.8	3.0 ± 3.3	0.56	3.2 ± 2.7	2.9 ± 3.3	0.54
Bowel preparation time (min)	351.0 ± 81.3	332.1 ± 81.5	0.10	350.4 ± 81.4	330.8 ± 82.5	0.10
Requirement of additional laxative	26 (24.8%)	26 (24.8%)	1.00	25 (25.3%)	25 (25.8%)	1.00

BBPS, Boston Bowel Preparation Score. Results are presented as mean ± standard deviation or number of patients.

When the bowel cleansing level was compared between the two groups among patients ≥70 years of age of the PP analysis set, the BBPS value for the whole colon was equivalent between the two groups (8.2 ± 0.9 vs. 8.2 ± 0.9, respectively; *P* = 0.95). The rates of patients achieving adequate bowel preparation were 95.5% in group A and 97.2% in group B without significant difference.

### Secondary endpoints

Table 2 also shows the bowel preparation time and the requirement for additional laxatives between the two groups. While the mean first defecation time was shorter in group A than in group B (4.5 ± 5.6 h vs. 6.7 ± 3.2 h, respectively; *P* < 0.001), bowel preparation time was no different between the two groups. Although 25 patients required additional laxatives in both groups, the rate did not differ between the groups.

Table 3 summarizes the procedure‐related outcomes. There were no significant differences between the groups with respect to cecal insertion time, withdrawal time, polyp detection rate, and cancer detection rate.

**Table 3:** Procedure‐related outcome

	Group A	Group B	*P* value
Achievement of total colonoscopy	99 (100%)	97 (100%)	1.00
Insertion time (min)	6.9 ± 4.4	6.2 ± 3.6	0.21
Withdrawal time (min)	8.2 ± 2.8	8.0 ± 2.3	0.60
Operator of colonoscopy, trainees	41 (41.1%)	32 (33.0%)	0.24
Polyp detection	45 (45.5%)	52 (53.6%)	0.31
Number of polyps	1.1 ± 1.6	1.2 ± 1.5	0.64
Location of polyp, right colon	32 (32.3%)	37 (38.1%)	1.00
Cancer detection	1 (1.0%)	5 (5.2%)	0.12

Results are presented as mean ± standard deviation or number of patients.

**Figure 4:**
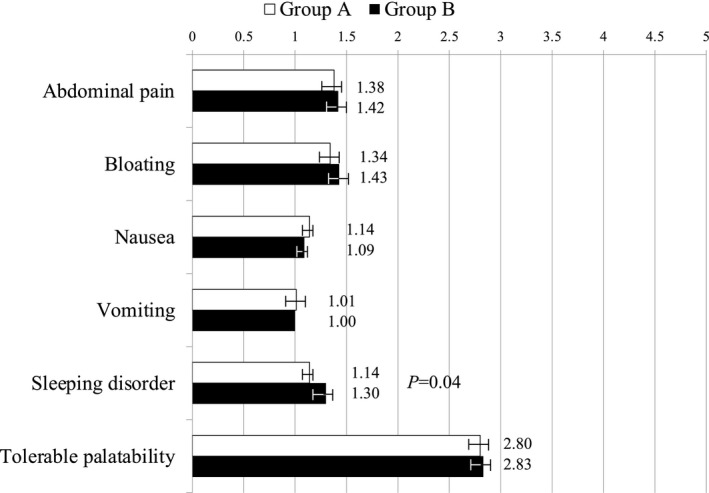
Patients’ tolerability and adverse events. Group A: elobixibat 10 mg + PEG‐Asc 1 L. Group B: sodium picosulfate 20 mL + PEG‐Asc 1 L. PEG‐Asc: polyethylene glycol formulation containing ascorbic acid. Results are presented as mean ± standard deviation or number of patients.

Figure 4 summarises the adverse events and palatability of bowel preparation in the two groups. While abdominal pain and bloating were the two most frequent adverse events, no significant difference was found in terms of gastrointestinal adverse events. In contrast, patients in group B complained of sleep disturbance more frequently than in group A (1.14 ± 0.43 vs. 1.30 ± 0.63, respectively; *P* = 0.04). The palatability score of the bowel preparation was comparable between the two groups.

## Discussion

Elobixibat is the first ileal bile acid transporter (IBAT) inhibitor. IBAT inhibitors block ileal absorption of bile acids by interrupting the enterohepatic bile circulation, resulting in a fall in serum cholesterol and an increase in bile acid secretion into the colon. The increase in colonic bile acids causes mucosal secretion and enhances colonic motility.^15^, ^16^, ^23^ It is well known that excessive bile acids in the colon by the disruption of the enterohepatic circulation in ileal disease or with resection can cause diarrhea.^25^, ^26^ By exploiting this function of bile acids, elobixibat is a potential treatment for chronic constipation. In addition, because elobixibat acts selectively in the gut, a lower incidence of adverse effects can be expected.^24^ In fact, the phase 3 trial in Japan demonstrated therapeutic efficacy and safety of 10 mg/day of elobixibat,^17^ and the medication was approved in January 2018 for the treatment of chronic constipation.

In the present study, BBPS value for the whole colon was equivalent between the two groups. Similar cleansing effect of the two bowel preparations was also shown when analysing at each colonic segment. Although the bowel preparation that can improve bowel cleansing level at the right colon seems to be ideal, the present study could demonstrate non‐inferiority of elobixibat plus 1‐L PEG‐Asc (group A) compared with sodium picosulfate plus 1‐L PEG‐Asc (group B) as bowel preparation for colonoscopy. In addition, sleep disturbance was less frequent in group A than in the group B, although no difference in the percentage of patients receiving sleep treatment was found between the groups. This difference could be mainly attributed to the difference in the timing of medication between elobixibat and sodium picosulfate because the latter medication must be taken the night before colonoscopy. Thus, it seems plausible that elobixibat is a better alternative to sodium picosulfate in combination with PEG‐Asc for bowel preparation of colonoscopy because the medication can be taken in the morning the day before colonoscopy.

While there was no difference in the number of defecations on the day before colonoscopy between the two groups, the first defecation time on the day before colonoscopy was significantly shorter in group A than in group B. Because elobixibat promotes defecation by increasing mucosal secretion and colonic motility,^15^, ^16^, ^23^ the medication might enhance colonic transit of feces more effectively compared with sodium picosulfate, which is a stimulant laxative. In addition, it often takes much longer for bowel preparation for colonoscopy in elderly patients who regularly use osmotic or stimulant laxatives.^27^ While bowel cleansing level was similar between the two groups, the mean first defecation time tended to be shorter in group A than in group B when analysed in elderly patients. Therefore, elobixibat might shorten the bowel preparation time for colonoscopy owing to its different mechanisms of action in elderly patients complicated by chronic constipation.

In the present study, the scores for all adverse events other than sleeping disorder did not differ between the two groups. The palatability score also did not differ between the two groups; however, it seems likely that these non‐significant results were mainly attributed to patients’ impression of PEG‐Asc. Therefore, it may have been better to assess the tolerability of bowel preparation on the day before and on the day of colonoscopy, separately. However, a significant difference in sleeping disturbance frequency between the two groups was obviously caused by the bowel preparation on the day before colonoscopy. The benefit of bowel preparation using elobixibat is that the medication in the morning on the day before colonoscopy encourages defecation during the day, thus reducing sleep disturbance on the day before the colonoscopy. Adverse events observed in the present study were considered mild, as in previous studies of PEG‐Asc.^2^, ^10^, ^20^ In terms of cost‐effectiveness, there was no large difference in drug costs between the two groups (group A, 2231.2 JPY vs. group B, 2264.8 JPY). With these considerations, it seems likely that bowel preparation with elobixibat plus 1‐L PEG‐Asc is more acceptable compared with that with picosulfate plus 1‐L PEG‐Asc.

The present study has several limitations. First, while bowel cleansing level did not differ between the two groups, some patients in both groups required additional laxatives. Therefore, it is possible that bowel cleansing levels shown in the present study did not necessarily represent the true bowel cleansing levels of both preparations. Second, determining the bowel cleansing levels under colonoscopy was subjective, which introduced bias in assessing the efficacy of bowel preparation. To minimize this bias, two endoscopists blinded to the type of bowel preparations reviewed the endoscopic images. Third, the present study compared the clinical usefulness of two types of bowel preparation; however, adverse events and tolerability in this study did not necessarily show a difference between elobixibat and sodium picosulfate.

In conclusion, considering the equivalent bowel cleansing effect and less frequent sleep disturbance of elobixibat plus 1‐L PEG‐Asc regimen, this method can be considered an alternative bowel preparation for colonoscopy.

## Conflict of Interest

Authors declare no conflict of interest for this article.

## Funding Information

None.

## Supporting information


**Appendix S1** The Boston Bowel Preparation Scale score (BBPS score).
**Appendix S2** Assessment of adverse events and palatability.

## Data Availability

The datasets used and/or analysed during the current study are available from the corresponding author on reasonable request.
