# Supplementary material for: Efficacy of elobixibat as bowel preparation agent for colonoscopy: Prospective, randomized, multi‐center study
Source: Dig Endosc. 2021 May 24;34(1):171–9. doi: 10.1111/den.14010 (PMC9290049; doi:10.1111/den.14010)
Supplement: Supplementary file 1 — Appendix S1 The Boston Bowel Preparation Scale score (BBPS score). Appendix S2 Assessment of adverse events and palatability. [file DEN-34-171-s001.docx]

**Supplementary material**

Appendix S1: The Boston Bowel Preparation Scale score (BBPS score)

The BBPS score was determined by the sum of a 4-point scoring system at each colonic segment (the right side of the colon, the transverse colon and the left side of the colon), with the total score ranging from 0 to 9. The colonic cleansing level of each segment was determined by the 4-point scoring system as follows: 0, unprepared colonic segment with mucosa not seen because of solid stool that cannot be cleared; 1, a portion of the mucosa of the colonic segment is visible, but other areas of the colonic segment are not clearly visible because of staining, residual stool and/or opaque liquid; 2, minor amount of residual staining, small fragments of stool and/or opaque liquid, but the mucosa of the colonic segment is clearly visible and 3, the entire mucosa of the colonic segment is clearly visible, with no residual staining, small fragments of stool or opaque liquid. Based on the BBPS, bowel cleansing level was categorised into four grades: excellent (BBPS 8–9), good (BBPS 6–7), poor (BBPS 3–5) or inadequate (BBPS 0–2). More than poor (BBPS score 6 or higher) was considered adequate bowel preparation.

Appendix S2: Assessment of adverse events and palatability

Patients were asked to assess the degree of abdominal pain, bloating, nausea, and vomiting using a 5-point scale (1 = none, 2 = mild, 3 = moderate, 4 = severe or 5 = very severe), and sleep disturbance using a 5-point scale (1 = no change, 2 = a little hard to sleep, 3 = hard to sleep, 4 = quite hard to sleep, or 5 = no sleep at all). More than 3 points were considered intolerable for bowel preparation. The palatability of the bowel preparation was graded into 5 categories (1= disgusting, 2 = unpleasant, 3 = acceptable, 4 = good or 5 = very good).
